# Shall the State Medical Association Have a Fixed Abode, and Place of Meeting; a State Medical Library

**Published:** 1887-12

**Authors:** 


### EDITORIAL DEPARTMENT.

F. E. DANIEL, M. D., EDITOR AND PUBLISHER

COLLABORATORS:

*E. J. Doering, M. D., Chicago.*

*Geo. Cupples, M. D., San Antonio.*

*Odo Betz, M. D., Germany.*

*E. J. Beall, M. D., Fort Worth, Texas.*

*T. C. Osborn, M. D., Cleburne, Texas.*

*Wm. Penny, M. D., New York.*

*H. O. Marcy, M. D., Boston.*

*C. K. Gregg, M. D., Mexico.*

*R. M. Swearingen, M. D., Austin.*

*C. H. Wilkinson, M. D., Galveston.*

*J. W. McLaughlin, M. D., Austin.*

*R. H. L. Bibb, M. D., Mexico.*

### SHALL THE STATE MEDICAL ASSOCIATION HAVE A FIXED ABODE AND PLACE OF MEETING?—THE ESTABLISHMLNT OF A STATE MEDICAL LIBRARY.

At the next meeting of the Texas State Madical Association, which will take place at Galveston, on the fourth Tuesday in April next, there will be presented for consideration, a new Constitution and By-Laws. The subject has been made the special order for the first day of the session.

There are many, and important changes needed; and from a casual reading of the new draft, as it will be presented, and as it appears in the present year's transactions, we see that an effort has been made to meet some of them; for instance; we pointed out in the issue of the JOURNAL (March, 1887), just preceeding the last meeting, the very astounding fact, that under our present Constitution and By-laws, *the minority virtually rules in all elections*, and other important matters, and that on no question is the voice of the majority heard.

This is because, under our present working, one representative only, from each county votes, no matter whether there be in that county, one physician, or one hundred; and that delegate has a voice in the election of all officers. To illustrate: Travis county has an organized society of forty members, and sends up eight delegates ; only one of those delegates goes into the Nominating Committee, and his vote, supposed to represent the voice of forty members, has no more weight in the choice of officers, than that of a delegate from the remotest county, in which there do not reside, perhaps, all told, ten physicians, and in which there may not be a single member of the State Association, or of any local organization. The committee propose to change this we believe; or, if they do not, it *should be* changed, and there should be some way provided, whereby the actual number of members of county societies shall be represented and heard ; or the actual number of physicians residing in a county. It should be so arranged that a delegate should cast a vote for every five members he represents, since he is selected on that basis. But, we instance this, as only one of the defects of our system—only one point whereon the Constitution and By-laws need amending.

No more important change in the By-laws could come up for consideration than that the Association should have "a local habitation," a place of permanent abode,—and of annual meeting ; and the establishment of a State Medical Library, should be considered also, in this connection.

The plan of having the Association meet at a different place each year, is, in our judgment, open to many and serious objections; while much can be said in favor of having a fixed abode, and a regular place of meeting. We will not stop to enumerate all the objections to the present plan, only pointing out that what is convenient to a given delegation this year, may be very inconvenient to it another, and that in consequence, there is, with the exception of a score of zealous members, who, to their credit, be it said, stop at no obstacles, but attend all meetings, far or near, convenient or otherwise, an entirely different set of men at each meeting. When we met at Houston, there was a large local representation ; and so with Austin and Dallas, and the more remote members, as a rule, did not attend ; hence the complexion of those conventions was entirely unlike. In brief, we seldom got the same set of men together ; the identify of one meeting is almost lost in the succeeding, and for the reasons stated. It requires no argument to show the importance of cohesion,—of assembling, year after year, the same men.

Now, if the Association had a fixed abode—say at the Capitol,—(and there are many and cogent reasons why the Capitol should be selected), sooner or later, every physician in good standing, within a radius of many miles, would become a member, and would attend the meetings with something like regularity ; while the distance to be traveled by other members, would not be greater than it is, occasionally, to a large number when the Association meets on the borders of the State, like at Galveston and Tyler, for example. The Legislature assembles in Austin each time; the courts are held there, and many other bodies , and why should a State Medical Association, made up of representatives from all parts of the State not do so ; instead of running here and there, and scattering, as they do ; for, take a member residing, say, in Fort Worth, and if it should happen that the Association should meet several times in succession at a great distance from Fort Worth, in all human probability that members will loose his identity with, and interest in the Association. We see this every year,—members drop out unless it is convenient to attend the meetings.

Much could be said on the head of doing away with the burden of entertainment being thrown on the local physicians ; the Association should provide its own entertainment, and if there were a neutral point, a permanent place of meeting, this would soon follow : but the main argument is in favor of permanency of the *archives*.

It is understood, that in the magnificent new Capitol now being built, a room or hall will be, or has, by act, been, set aside for the Medical profession ! and an effort is now being made by Austin physicians to secure such room. In this room should be kept the Secretary's office and the archives of the Association, and a library should be established. To show further the importance of some such change, and without in ending, in the least to reflect on the former Secretaries of the Association,—for a part of the fault to be mentioned is the result of the migratory habit of the Association—there is nothing like a complete *record* of the Association and its doings, in possession of that body ! Prior to 1878, we have *understood* that the Transactions were published in book form. There is not in the Secretary's office, a single copy ; from 1878 to 1883, we have *understood*, the Transactions were printed in the *Galveston*

*Medical Record.* There is not a copy in the Secretary's office ! In 1883 volume Transactions was resumed. There is, in the Secretary's office *one single copy* of that year, in bad condition, and paper bound. Since 1883 there is a full file of all proceedings ; the Transactions of 1884 and 1887, being in paper, and those of 1885 and 1886, being in cloth. (We have had the file copy of 1887 and of 1884 bound in cloth.) If there are any papers or documents of any kind, other than the Secretary's minutes, in an old book, relating to the history or proceedings of the Association prior to the Tyler meeting in 1883, they are not to be found in the Secretary's office. In fact, —heretofore the Secretary's "office" seems to have been a desk, only, and all that there is of record of the Association that came into the hands of the present Secretary is (or was) in that desk ! We will venture that there are a dozen libraries in America which have a better record of the Texas State Medical Association, than is in possession of the Secretary.

With regard to a library, if the Association were to change the plan of itineracy and make Austin the abode of the Society, a library could and would soon be established. The Secretary receives in exchange the Transactions of other Associations and of Health Boards, besides numerous pamphlets. These should be preserved, catalogued and cared for ; and the record of the Association might possibly be perfected by obtaining the missing records from some generous members. This JOURNAL offers here and now, to donate to such library, if started, all the Medical Journals and monograms and pamphlets that come in exchange, and there are perhaps fifteen to twenty-five hundred volumes (pamphlets), each year ; besides a number of the newest, latest and best standard works, that are sent us for "review," as fast as they issue from the Medical publishing houses. There are many of such books which we will cheerfully donate to a library for the State Medical Association, if any such arrangement is made as has been outlined above. Doubtless others would also donate books and pamphlets.

Then the Association, meeting in this hall each year, every member would have access to all documents of record, volumes of Transactions, as well as to all the current and standard Medical literature of the day.

In after years, when the Medical history of Texas shall be written, the archives of the Texas State Medical Association should be a mine of useful information. There is a fearful hiatus now existing, and it should be closed, while it is yet possible, may be, to procure some of the missing records.

The establishment of a State Medical Library ought to be a cherished aim of this Society. Every document or publication that relates to epidemic diseases or sporadic diseases that prevail, or have prevailed in Texas, should be carefully preserved in permanent form, and made available to the future Medical historian.

This is the more imperative now, since the same thing has been done, to a considerable extent, with reference to the surgery of the past,—by our indefatigable committee. The same hall used for the library could be used also for the annual meetings of the State Association.

Should this change be made in our Constitution and By-laws, and the future meetings of the Association be held at Austin in their own library hall,—we predict a great impetus would be given *organization*, and original scientific research ; a new era would dawn on "Texas Medicine."

The President of the Louisiana State Medical Association is advocating a similar change in that body, and we commend his able address to the profession of Louisiana, and the above remarks to the careful consideration of our members, and especially to that of our calm, far seeing and progressive President, Sam R. Burroughs.

At the last meeting of the State Association, the Secretary, in his report, pointed out the absence from his office of all printed record anterior to '83, and the necessity of providing some means for the care and preservation of the mass of literature, now accumulating; but no action was taken. A resolution should be passed authorizing the Secretary to advertise for, and otherwise endeavor to procure the missing volumes of Transactions, and of the *Medical Record*, and, if need be, an appropriation should be made to pay for them, if they can be found.

